# Supplementary material for: Fasoracetam in adolescents with ADHD and glutamatergic gene network variants disrupting mGluR neurotransmitter signaling
Source: Nat Commun. 2018 Jan 16;9:4. doi: 10.1038/s41467-017-02244-2 (PMC5770454; doi:10.1038/s41467-017-02244-2)
Supplement: Supplementary file 1 — Supplementary information [file 41467_2017_2244_MOESM1_ESM.pdf]

Supplemental Figure 1

A

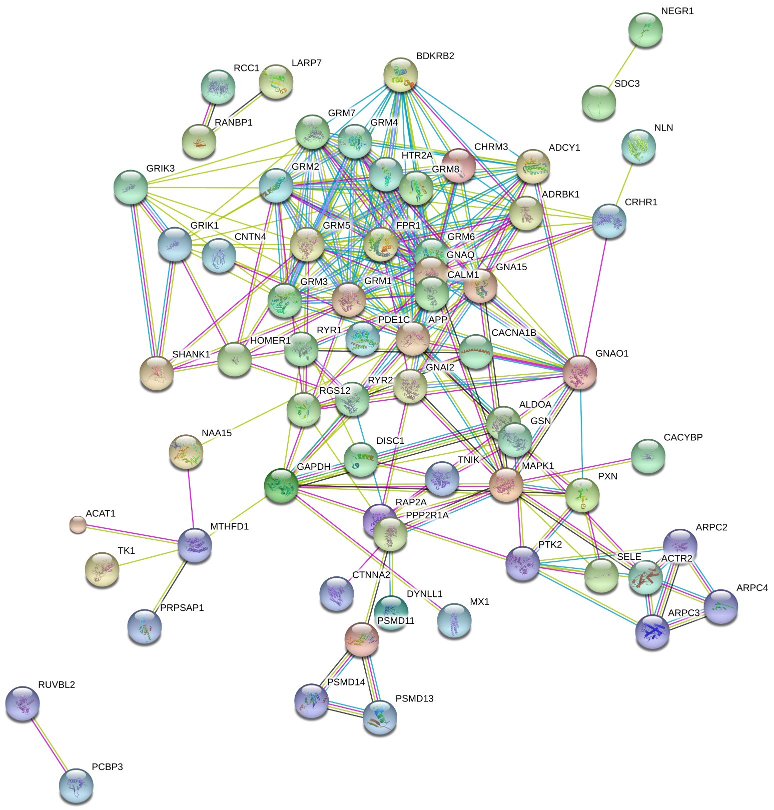

Network graph depicting pairwise experimentally validated and computationally predicted pairwise interactions across the Tier 1 mGluR genes as identified by StringDB

B

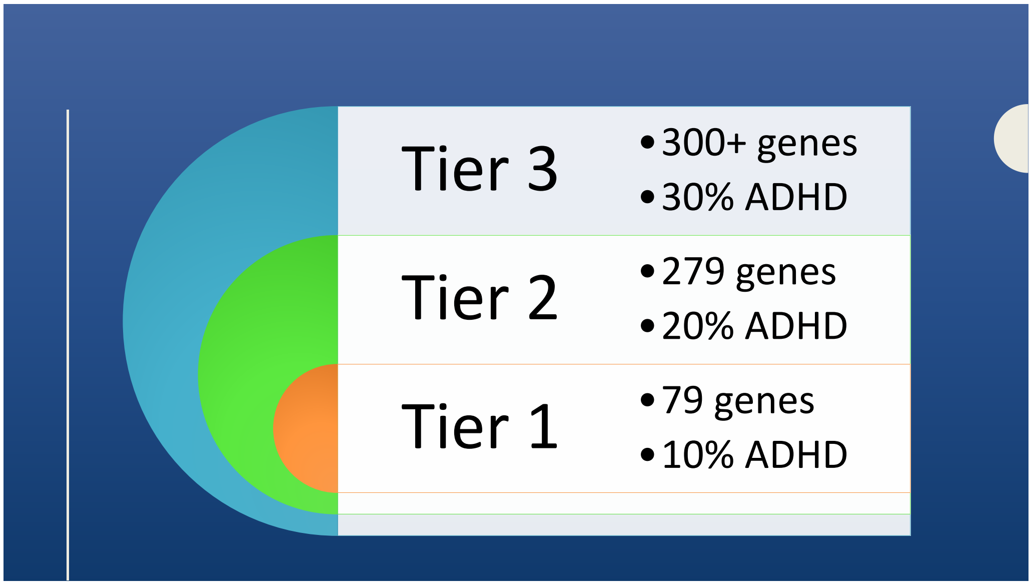

The three tiers of mGluR network genes

Supplemental Figure 2

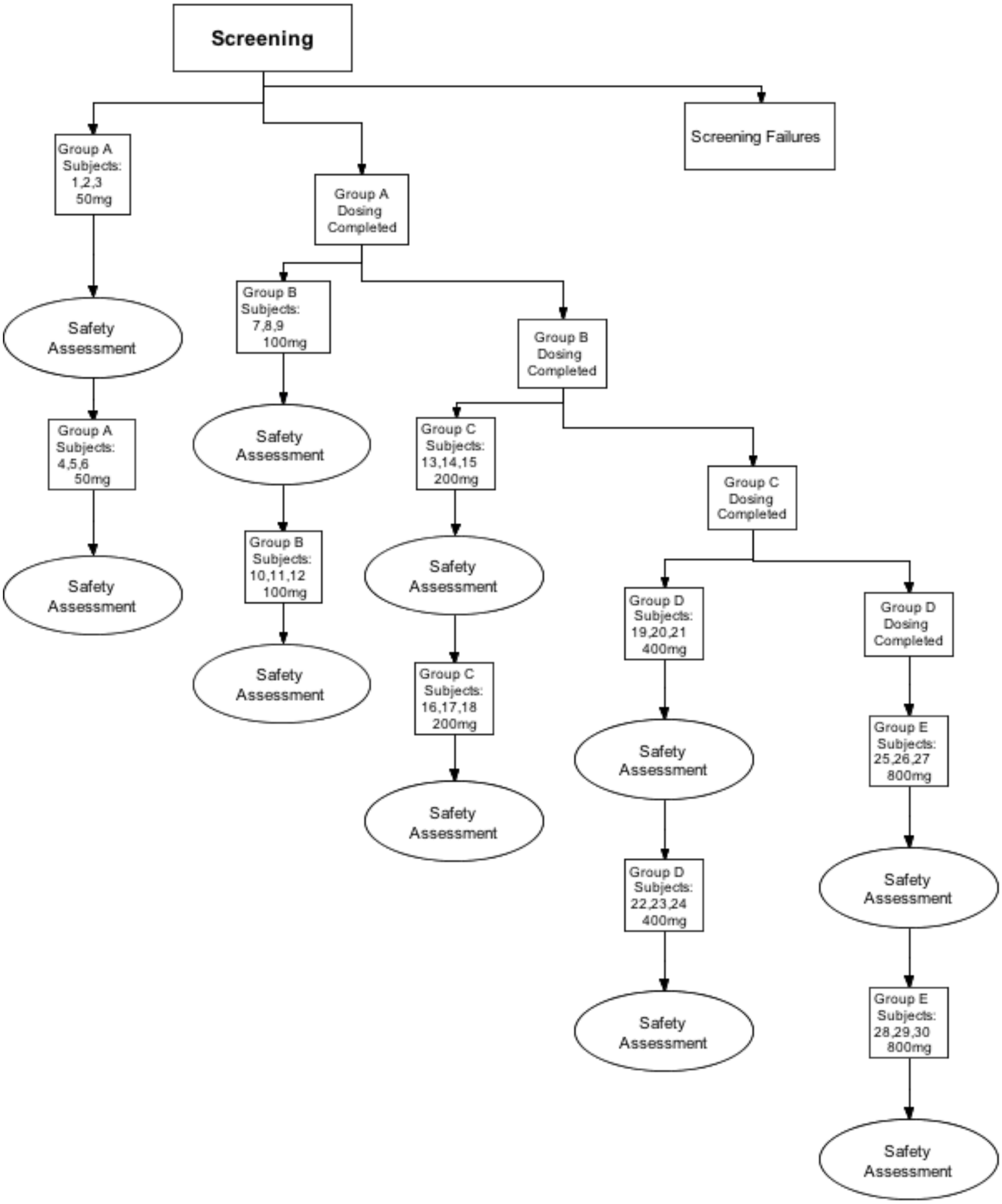

Flow diagram illustrating the dose-escalation and subject enrollment protocol study design

Supplemental Figure 3

Week of study	Subjects/Doses					
1	PK<br>1,2,3<br>50mg					
2		PK<br>4,5,6<br>50mg				
3	DE<br>1,2,3<br>Placebo	DE<br>4,5,6<br>Placebo	PK<br>7,8,9, 10,11,12<br>100mg			
4	DE<br>1,2,3<br>50mg bid	DE<br>4,5,6<br>50mg bid	DE<br>7,8,9,10,11,12<br>Placebo	PK<br>13,14,15,16,17,18<br>200mg		
5	DE<br>1,2,3<br>100mg bid	DE<br>4,5,6<br>100mg bid	DE<br>7,8,9,10,11,12<br>50mg bid	DE<br>13,14,15,16,17,18<br>Placebo	PK<br>19,20,21,22,23,24<br>400mg	
6	DE<br>1,2,3<br>200mg bid	DE<br>4,5,6<br>200mg bid	DE<br>7,8,9,10,11,12<br>100mg bid	DE<br>13,14,15,16,17,18<br>50mg bid	DE<br>19,20,21,22,23,24<br>Placebo	PK<br>25,26,27,28,29,30<br>800mg
7	DE<br>1,2,3<br>400mg bid	DE<br>4,5,6<br>400mg bid	DE<br>7,8,9,10,11,12<br>200mg bid	DE<br>13,14,15,16,17,18<br>100mg bid	DE<br>19,20,21,22,23,24<br>50mg bid	DE<br>25,26,27,28,29,30<br>Placebo
8			DE<br>7,8,9,10,11,12<br>400mg bid	DE<br>13,14,15,16,17,18<br>200mg bid	DE<br>19,20,21,22,23,24<br>100mg bid	DE<br>25,26,27,28,29,30<br>50mg bid
9				DE<br>13,14,15,16,17,18<br>400mg bid	DE<br>19,20,21,22,23,24<br>200mg bid	DE<br>25,26,27,28,29,30<br>100mg bid
10					DE<br>19,20,21,22,23,24<br>400mg bid	DE<br>25,26,27,28,29,30<br>200mg bid
11						DE<br>25,26,27,28,29,30<br>400mg bid

Summary flowchart of study implementation week by week including dose escalation and study subject group. PK: pharmacokinetic; DE: dose escalation; bid: twice a day

Supplemental Figure 4

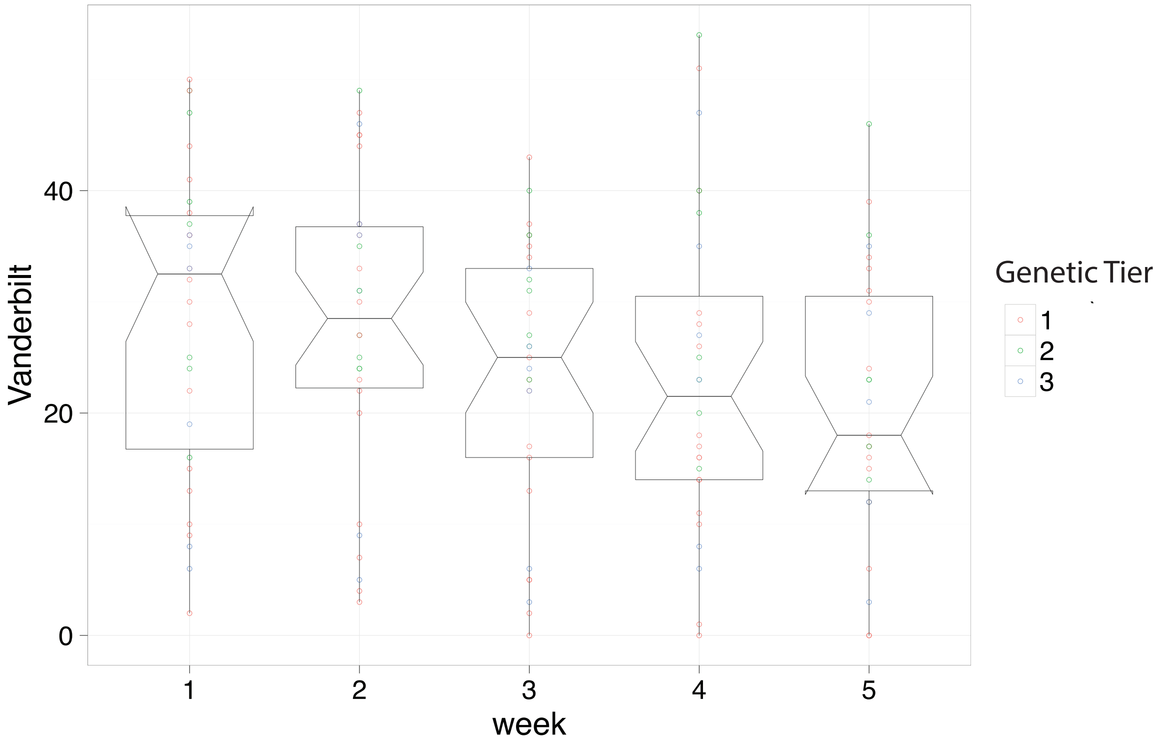

Box-plot of Vanderbilt scores for all study subjects across the five weeks of the study stratified by the patient's genetic tier. Each dot represents one subject's score. Center line in each box is the median.

Supplemental Figure 5

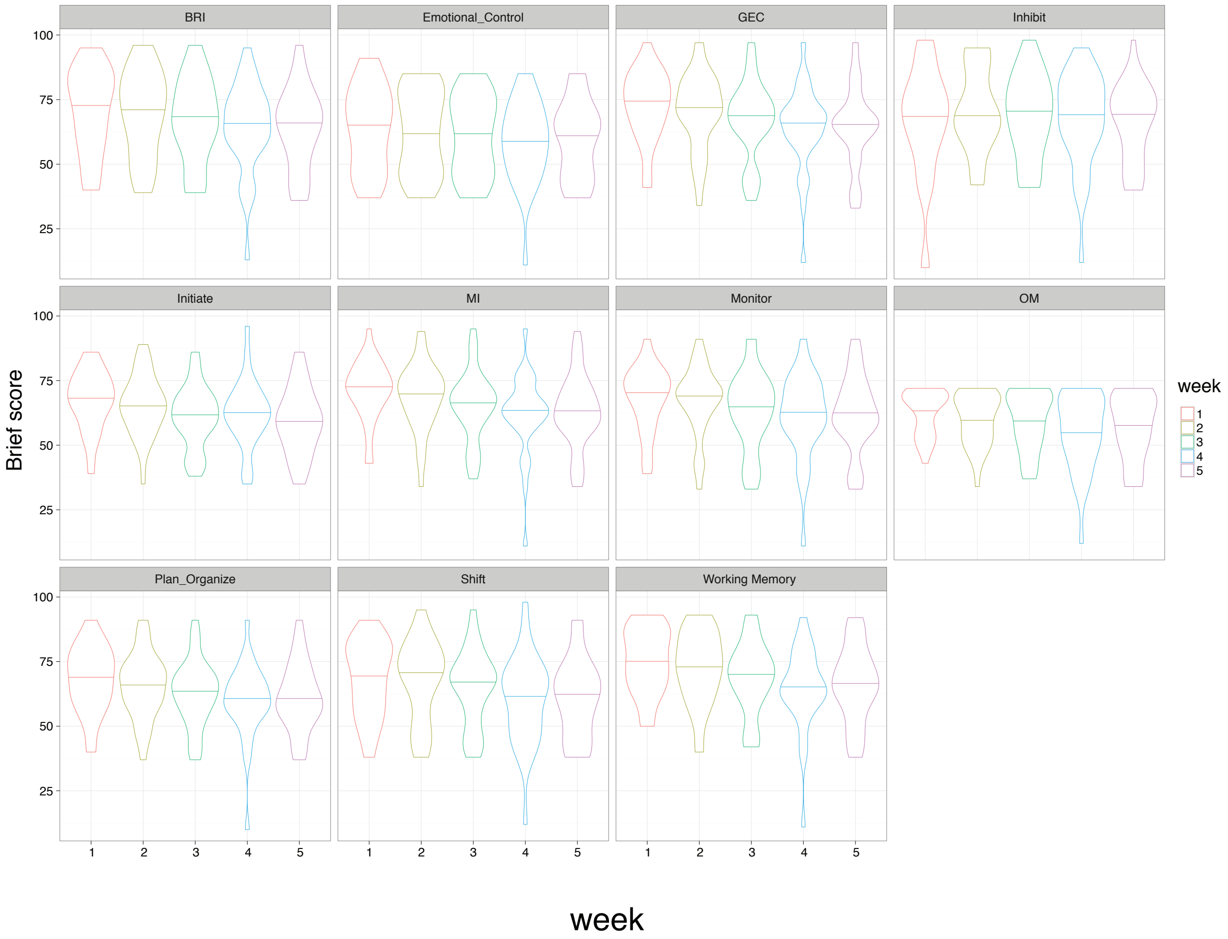

Violin plots of each of the Brief Score sub-categories by study week. Center line denotes the median score.

Supplemental Tables

Supplemental Table 1: Study flowsheet – ENROLLMENT and PK

	S	E	**Time after first NFC-1 dose, in hours**											
			PK-Day 1											PK-Day 2
			Pre-dose	0	0.5	1.0	1.5	2	3	4	6	8	12	24
Genomics Informed Consent/Assent	X													
Genotyping for GRM-genes	X													
Telephone Screen[A]	X													
Informed Consent/Assent		X												
K-SADS		X												
WAIS Brief IQ (or use past testing)		X												
BRIEF (Parent; Self)		X												
Quotient®ADHD test		X	X											X
PERMP-Math Baseline Test		X												
PERM-Math Test			X											X
Actigraphy (continuous)			X	X	X	X	X	X	X	X	X	X	X	X
CGI-I & CGI-S		X												
Demographics/Medical History		X												
Physical Exam, Weight & Height		X	X											X
Vital Signs: BP, HR, RR		X	X					X		X	X		X	X
12-lead ECG		X	X											X
Hematology & clinical chemistry			X											
Urinalysis			X											
Urine βhCG test (menst. females)			X											
Meals								X			X		X	X
NFC-1 administration (on-site)				X *										
Blood sampling for PK			X		X	X	X	X	X	X	X	X	X	X
Adverse Event Monitoring (including suicidal ideation/plan)			X	X	X	X	X	X	X	X	X	X	X	X
Dispense Week 1 medication														X

S: Screening; E: Enrollment: ECG: Electrocardiogram; PK: Pharmacokinetics ; BP: blood pressure; HR: Heart Rate; RR: respiratory rate; hCG: human chorionic gonadotropin

Supplemental Table 2: study flowsheet: Dose-escalation (1-14 days after 24H PK study)

	Week 1	Week 2	Week 3	Week 4	Week 5	Week 9
	**Day 7 (±2)**	**Day 14 (±2)**	**Day 21(±2)**	**Day 28 (±2)**	**Day 35 (±2)**	**Day 75 (±2)**
Adverse event monitoring (Pittsburgh Side effects Rating Scale + suicidal ideation/plan)	X	X	X	X	X	phone
Laboratory Safety Tests (blood and urine)[A]	X	X	X	X	X	
Physical Examination	X	X	X	X	X	
Vital Signs: BP, HR, RR	X	X	X	X	X	
Body Weight (all points) & Height (week 1 only)	X	X	X	X	X	
12-lead ECG	X	X	X	X	X	
Urine 🚲hCG test (menstruating females only)	X	X	X	X	X	
Contraception verification (selected females)	X	X	X	X	X	
Vanderbilt Parent Rating Scale	X	X	X	X	X	
BREIF (Parent; Self)	X	X	X	X	X	
Quotient®ADHD test	X	X	X	X	X	
PERMP-Math test	X	X	X	X	X	
Actigraphy (continuous monitoring)	X	X	X	X	X	
CGI-S & CGI-I	X	X	X	X	X	
Dispense study drug[B]	X	X	X	X		
NFC-1 or placebo administration at home[C]	Placebo bid	50 mg bid	100 mg bid	200 mg bid	400 mg bid	
Retrieve pill bottle/pill count	X	X	X	X	X	

A: Blood draws for hematology (RBC, WBC with differential, platelet count) and clinical chemistry (electrolytes, albumin, ALT, AST, alkaline phosphatase, bilirubin, BUN, creatinine kinase, glucose), NFC-1 and major metabolite serum levels.
B: Study drug for Week 1 administered at end of PK study; study drug for next week dispensed at each clinic visit
C:Dose escalations to be determined by CGI-S and CGI-I scores at end of each week of treatment; maximum doses indicated

Supplemental Table 3: Detailed adverse events reported by study week

AE	Enrollment	PK/day2	Week 1	Week 2	Week 3	Week 4	Week 5	Week 6 Followup	Week A - Unscheduled Visit	Week B - Unscheduled Visit	Total
**Abdominal Discomfort**						1					**1**
**Abdominal Pain**							1				**1**
**Abdominal Pain Upper**	1	1	1	1	1	3	3	2			**13**
**Agitation**						1					**1**
**Alopecia**								1			**1**
**Anger**				1							**1**
**Anxiety**								3			**3**
**Back Pain**						1					**1**
**Blepharospasm**								1			**1**
**Blood creatine phosphokinase increased**					1						**1**
**Chills**		1									**1**
**Contusion**	1	1		1		1					**4**
**Cough**				1	2	1					**4**
**Decreased Appetite**			1								**1**
**Depressed Mood**				1		1		2			**4**
**Depression**								1			**1**
**Dermatillomania**		1									**1**
**Diarrhoea**		1	3		1	1	1	2	1		**10**
**Dizziness**		1	1		2	2	3	1			**10**
**Dry eye**					1						**1**
**Dysmenorrhoea**						1					**1**
**Ecchymosis**		1									**1**
**Enuresis**			1	1							**2**
**Epistaxis**	1		1								**2**
**Eye pain**			1								**1**
**Eye Pruritus**						1					**1**
**Fatigue**		5	7	3	6	3	1	1	1		**27**
**Flatulence**		1									**1**
**Flushing**				1							**1**
**Head Injury**					1						**1**
**Headache**		2	12	9	6	8	3	4			**44**
**Hyperhidrosis**							1				**1**
**Hypertension**								1			**1**
**Impetigo**				1	1						**2**
**Increased Appetite**				1							**1**
**Insomnia**								1			**1**

**Irritability**			1	1		2	1	4			**9**
**Laceration**		2				1					**3**
**Malaise**				1							**1**
**Memory Impairment**					1			1			**2**
**Migraine**					1						**1**
**Mood Altered**			1								**1**
**Mood swings**								1			**1**
**Muscle Spasms**							1				**1**
**Muscle Twitching**								1			**1**
**Nasal Congestion**					1						**1**
**Nasal Discomfort**			1								**1**
**Nausea**	1	3				2	1				**7**
**Onychophagia**			2		1						**3**
**Oropharyngeal Pain**		1				1	2				**4**
**Pain**				1							**1**
**Pain in Extremity**										1	**1**
**Presyncope**					1						**1**
**Pyrexia**				1		1	1	2			**5**
**Rash**						1					**1**
**Restlessness**								1			**1**
**Rhinorrhoea**						1					**1**
**Self-injurious Ideation**					1						**1**
**Skin Irritation**		1									**1**
**Social Avoidant Behaviour**			1					1			**2**
**Somnolence**			2		1						**3**
**Swelling**					1						**1**
**Swelling Face**		1									**1**
**Tearfulness**				3							**3**
**Thinking Abnormal**					1						**1**
**Tinnitus**							1				**1**
**Visual Impairment**			1				1				**2**
**Vomiting**								1		1	**2**
Total	***4***	***23***	***37***	***28***	***32***	***34***	***21***	***32***	***2***	***2***	***215***

Note that week 1 refers to study baseline or a week of placebo and each week between weeks 2-5 the patient received the study drug

Supplemental Table 4

Medications Discontinued at Enrollment and those maintained during the study

**Study participant**	**Stimulants**	**Atomoxetine**	**Guanfacine**	**Other Meds**	**During Study**
101				Fluoxetine	
102				Clonidine-sleep	Clonidine
104	Vyvanse				
105	Adderall				
107	Vyvanse				
108				Lamictal	Lamictal
109		**Atomoxetine**			
113				Sertraline	Sertraline
114	Concerta				
115				Clonidine (sleep)	
118	Concerta				
119	Concerta				
121				Clonidine (sleep)	
125				Risperidone	

Note: Unless a medication appears in the last column "During Study" all other medications were discontinued for the duration of the washout period.
