# Supplementary material for: Fasoracetam in adolescents with ADHD and glutamatergic gene network variants disrupting mGluR neurotransmitter signaling
Source: Nat Commun. 2018 Jan 16;9:4. doi: 10.1038/s41467-017-02244-2 (PMC5770454; doi:10.1038/s41467-017-02244-2)
Supplement: Supplementary file 3 — Description of Additional Supplementary Files [file 41467_2017_2244_MOESM3_ESM.pdf]

**Description of Additional Supplementary Files**

File Name: Supplementary Data 1
Description: This file includes 600 mGluR network genes that may have similar relevance to ADHD as the 79 genes we have shown to be significantly enriched in ADHD. However, we have not confirmed enrichment of CNVs in these genes in patients with ADHD. Arranged in order are: Tier-1, 2 and 3 mGluR network genes previously shown to be enriched for Copy Number Variation (CNVs) in patients with ADHD.
